# Structural Identification of *O*-Linked Oligosaccharides Using Exoglycosidases and MS^n^ Together with UniCarb-DB Fragment Spectra Comparison

**DOI:** 10.3390/metabo2100648

**Published:** 2012-10-04

**Authors:** Liaqat Ali, Diarmuid T. Kenny, Catherine A. Hayes, Niclas G. Karlsson

**Affiliations:** Department of Medical Biochemistry, Institute of Biomedicine, University of Gothenburg, 40530 Gothenburg, Sweden

**Keywords:** mass spectrometry, exoglycosidases, mucin, glycomics

## Abstract

The availability of specific exoglycosidases alongside a spectral library of *O*-linked oligosaccharide collision induced dissociation (CID) MS fragments, UniCarb-DB, provides a pathway to make the elucidation of *O*-linked oligosaccharides more efficient. Here, we advise an approach of exoglycosidase-digestion of *O*-linked oligosaccharide mixtures, for structures that do not provide confirmative spectra. The combination of specific exoglycosidase digestion and MS^2^ matching of the exoglycosidase products with structures from UniCarb-DB, allowed the assignment of unknown structures. This approach was illustrated by treating sialylated core 2 *O*-linked oligosaccharides, released from the human synovial glycoprotein (lubricin), with a α2–3 specific sialidase. This methodology demonstrated the exclusive 3 linked nature of the sialylation of core 2 oligosaccharides on lubricin. When specific exoglycosidases were not available, MS^3^ spectral matching using standards was used. This allowed the unusual 4-linked terminal GlcNAc epitope in a porcine stomach to be identified in the GlcNAc1-4Galβ1–3(GlcNAcβ1-6)GalNAcol structure, indicating the antibacterial epitope GlcNAcα1–4. In total, 13 structures were identified using exoglycosidase and MS^n,^ alongside UniCarb-DB fragment spectra comparison. UniCarb-DB could also be used to identify the specificity of unknown exoglycosidases in human saliva. Endogenous salivary exoglycosidase activity on mucin oligosaccharides could be monitored by comparing the generated tandem MS spectra with those present in UniCarb-DB, showing that oral exoglycosidases were dominated by sialidases with a higher activity towards 3-linked sialic acid rather than 6-linked sialic acid.

## Abbreviations

Fuc: fucose
Hex: hexose
HexNAc: *N*-acetylhexosamine
HexNAcol: *N*-acetylhexosaminitol
NeuAc: *N*-acetylneuraminic acid
GlcNAc: *N*-acetylglucosamine
GalNAc: *N*-acetylgalactosamine
PGM: porcine gastric mucin
LC: liquid chromatography
MS: mass spectrometry
MS^n^: tandem mass spectrometry
SDS-AgPAGE: sodium dodecyl sulfate-agarose/polyacrylamide composite gel electrophoresis
SDS-PAGE: sodium dodecyl sulfate-polyacrylamide gel electrophoresis
DTT: dithiothreitol
IAA: iodoacetamide
PVDF: polyvinylidene fluoride
Le^a^: lewis a
Le^x^: lewis x

## 1. Introduction

The oligosaccharide epitopes of cell surface proteins such as glycoproteins, glycolipids and proteoglycans have been considered as mediators for signal transduction from the outside environment to the inside of the cell [1]. The introduction of microbes and pathogens alter the expression of these oligosaccharide epitopes due to altered signal transduction [2]. This is due to the enzymatic modification of glycans triggered by signal transduction. However, in order to better understand the interaction of the cell with the outside environment and to establish a relationship between glycan structure and function, the glycomic investigation of cell surface proteins is essential.

Due to the macro and micro heterogeneity associated with *O*-linked glycans, glycomic analysis requires a combination of techniques such as exoglycosidases, lectins, mass spectrometry (MS) and NMR [3]. Exoglycosidase digestion is usually used to monitor the enzymatic modification and to reduce the complexity by cleaving the larger oligosaccharides into smaller units as well as to assign the structure and provide linkage specific information [4,5]. Increased sensitivity combined with detailed high throughput structural characterization of oligosaccharides is now possible using mass spectrometry [6,7,8]. Mass spectrometry has been applied to the structural elucidation of a number of biomolecules including oligosaccharides and has emerged as the premier technique for glycan characterization in various biologically important molecules [9,10]. Mass spectrometry offers distinct advantages because of its sensitivity and capability for obtaining structure information through tandem MS. Tandem MS involves the isolation of specific ion species that are further examined for structural elucidation [8]. This allows the characterization of previously uncharacterizable oligosaccharides from natural glycoproteins by analysis of degradation products from specific exoglycosidase treatment [11]. However, the identification of oligosaccharide linkages posed tremendous challenges to mass spectrometry. 

Exoglycosidase digestion, either sequentially or in arrays is usually suggested for generating linkage information as well as for glycan characterization [5,12]. For *N*-linked oligosaccharides, these methods are well established. The nature of the heterogeneous *O*-linked glycosylation present in highly glycosylated mucin domains [2] and difficulties in labeling released *O*-linked oligosaccharides [13,14,15] makes LC-MS, in combination with exoglycosidases, an obvious choice for detecting and identifying the effect of exoglycosidases on heterogeneous mixtures. Using LC-MS^2^, the oligosaccharide sequences before and after the digestion the linkages between the individual glycan moieties can be monitored from the pattern of observed glycan fragments and the specificities of the exoglycosidases. The process of annotation of the resulting MS^2^ spectra is made quicker due to the development of UniCarb-DB, an LC/MS^2^ database of annotated *N-* and *O*-glycan structures [16]. The database provides mass spectrometric structural assignment of structures, which is based on LC/MS^2^ fragmentation. The database contains extensive information about glycan analysis including their HPLC details such as column types, solvents, gradients, flow rates and MS details such as modification, mode of detection, data acquisition and the type of devices used during analysis. In addition, the database provides MS^2^ spectra and annotated MS^2^ peak list of the identified structures. This allows a parent mass to be searched for and the comparison of the MS^2^ spectra of these known spectra to be compared to experimental data, therefore, reducing the necessity of manual annotation of glycan data analysis.

A previous study has shown a successful strategy of combined exoglycosidase digestion and MS^2^ spectral matching of *N*-linked oligosaccharides [17]. In the present study, *O*-linked oligosaccharides from human synovial lubricin, mucin from porcine gastric stomach and salivary glycoproteins (MUC5B and MUC7) was spectral matched with spectra from UniCarb-DB. The lack of confirmative matches in the database triggered within the sample an exoglycosidase treatment, wherein the structure of the generated product could again be subjected to spectral matching. The specificity of the exoglycosidase used allowed the identification of the oligosaccharide sequence of the substrate. It was also investigated how MS^n ^could be used to identify non-reducing monosaccharide units, where the lack of specific exoglycosidases prevented them to be removed. 

## 2. Results

**Figure 1:**
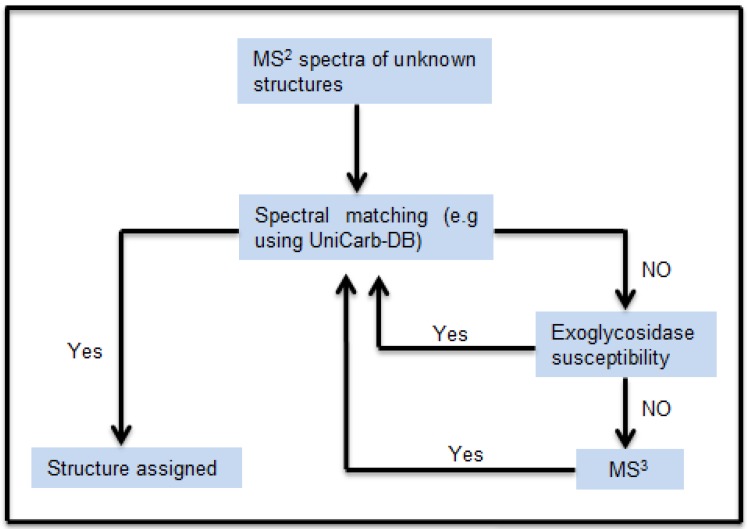
Schematic workflow for structural assignment of *O*-glycans using MS^n^ spectral match. The MS^2^ peak list of the isolated chromatographic peak were compared and scored with the peak list of the structure reported in the MS^2^ database UniCarb-DB. Poor scoring resulted in exoglycosidase digestion and concomitant scoring of MS^2^ spectra of the generated product. In absence of successful exoglycosidase digestion, MS^3^ were generated for spectral comparison.

The schematic workflow in Figure 1 shows how the MS^2^ peak list (*m/z* and relative intensity) of the isolated chromatographic peaks were compared with the MS^2^ peak list of the structures reported in the MS^2^ glycomic database UniCarb-DB. The structures, in particular sialylated structures, which did not give a good match, were exoglycosidase digested (in particular de-sialylated). The MS^2^ peak list of the exoglycosidase products generated were again compared with the MS^2^ peak list of the structures reported in the MS^2^ database UniCarb-DB. For structures wherein a specific exoglycosidase was lacking, an MS^3^ approach was used. The MS^3^ peak lists of unknown structures were compared with the MS^2^ peak lists from the UniCarb-DB database (if fragments were Y-ions), or compared MS^3^ spectra of fragments generated from standards. 

### 2.1. Investigation of Sialylated Structures in Human Synovial Lubricin

**Figure 2:**
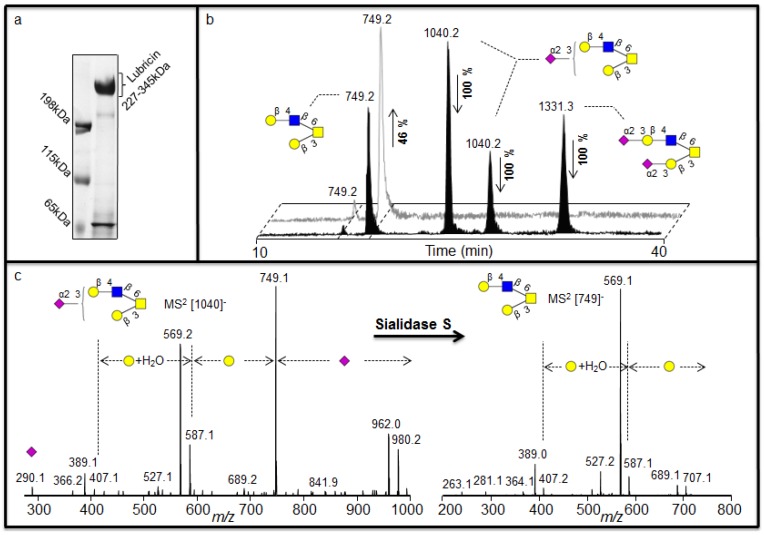
Negative ion LC-MS^2^ analysis of sialylated structures in human synovial lubricin. (a) Enrichment of human synovial lubricin by SDS-PAGE. (b) Selected ion chromatogram (SIC) of the [M - H]^-^ ions at *m/z* 749*,* 1040 and 1331 before (front) and after the treatment (back) with sialidase S confirming the α2-3 linked sialic acid configuration of oligosaccharide. Percentage shows the increase or decrease of the structures due to treatment. (c) MS^2^ spectrum of the [M - H]^-^ ions at *m/z* 1040 with α2-3 linked sialic acid before treatment and MS^2^ spectrum of the [M - H]^-^ ions at *m/z* 749 increased after the treatment. For explanation of symbols, see legend in Table 1

Negative ion LC-MS^2^ has been shown to provide detailed structural information of neutral oligosaccharides [8], but it has been suggested that linkage specific sialidases should be used to increase the information about sialylated oligosaccharides [18], where their MS^2^ spectra is less informative. The sequence and configuration of sialylated structures were addressed using human synovial lubricin oligosaccharides. The human synovial lubricin was isolated by SDS-PAGE (Figure 2a) and the oligosaccharides from the dominating band in the gel (227-345 kDa) were released by reductive β-elimination [8]. The coomassie stained gel also highlighted two additional bands in the regions of 200 kDa and 65 kDa. The band around 200 kDa regions was found to be fibronectin while the band at 65 kDa region was C terminus of lubricin when analyzed by proteomic means. These results have been published previously [19]. The spectra of the released oligosaccharides were dominated by mono- and di-sialylated structures when analyzed by LC-MS^2^. The assignment of the sialylated structures *i.e.* [M - H]^-^ ions at *m/z* 1331 (NeuAc_2_Hex_2_HexNAc_1_HexNAcol) and *m/z* 1040 (NeuAc_1_Hex_2_HexNAc_1_HexNAcol) gave indecisive scoring (R^2^) about the sequence of the structures (Table 1) when their MS^2^ spectral intensities were compared with spectra reported in the MS^2^ database UniCarb-DB [16]. The reason was that the sialylated structures gave similar R^2^ value between 1^st^ and 2^nd^ ranked structure as shown in Table 1. In addition, the MS^2^ spectra of the sialylated structures are less informative due to loss of labile sialic acid, which also made their assignment difficult. The less informative MS^2^ spectrum of the sialylated structures may also be the reason why they are not well assigned by spectral match. The table also shows the additional data from samples analyzed in this report. Overall it was indicated that neutral structures scored better than sialylated. This is illustrated by the differences in score between the best assigned as 1^st^ ranked (highest R^2^ value close to 1) and 2^nd^ ranked structure (2^nd^ highest R^2^). Therefore, it was concluded that once the sialic acid is removed by sialidase treatment, the remaining structure could be easily assigned by spectral matching. These data suggest that the quality of the spectra from sialylated structures only have limited information about the sequence beside the presence of terminal sialic acid. 

For sequence and configuration elucidation of sialylated structures, the released oligosaccharides of human synovial lubricin were incubated with sialidase S (*Streptococcus pneumonia*) specific for α2-3 linked sialic acid. After 16 h incubation, a complete degradation of the [M - H]^-^ ions at *m/z* 1040 (NeuAc_1_Hex_2_HexNAc_1_HexNAcol) and [M - H]^-^ ions at *m/z* 1331 (NeuAc_2_Hex_2_HexNAc_1_HexNAcol) (Figure 2b) could be shown, accompanied with an increase in the intensity of the [M - H]^-^ ions at *m/z* 749 (Hex_2_HexNAc_1_HexNAcol (Figure 2b), indicated that this was the exoglycosidase product generated after removal of sialic acid from the substrate. The MS^2^ spectral intensity correlation analysis of the [M - H]^-^ ions at *m/z* 749 with spectra reported in the MS^2^ database UniCarb-DB suggests that this was a core 2 structure with Galβ1-3(Galβ1-4GlcNAcβ1-6)GalNAc configuration (Table 1) which can be terminated with one sialic acid (on either of the branches) and with two sialic acid (on both branches). The complete degradation of the [M - H]^-^ ions at *m/z* 1331 and *m/z* 1040 indicated that the NeuAc moiety in both the structures are α2-3 linked (Figure 2b) and the MS^2^ spectral intensity correlation analysis of the structure created after the treatment (i.e [M - H]^-^ ions at *m/z* 749) further extended the assignment of the structure to be Galβ1-3(Galβ1-4GlcNAcβ1-6)GalNAcol (Table 1). The intensity of the product (i.e only 46%) did not increase proportionally to the decrease of the substrates due to differences in ionization efficiency. The complete degradation of the sialylated core 1 with [M - H]^-^ ions at *m/z* 675 (NeuAc_1_Hex_1_HexNAcol) could also be observed. This indicated that the NeuAc moiety is α2-3 linked to the Galβ1-3GalNAc α1-Ser/Thr sequence of the core 1 structure when the MS^2^ spectra of the structure ([M - H]^-^ ions at *m/z* 384) created after the treatment were compared with spectra reported in the MS^2^ database UniCarb-DB (Table 1). 

**Figure 3:**
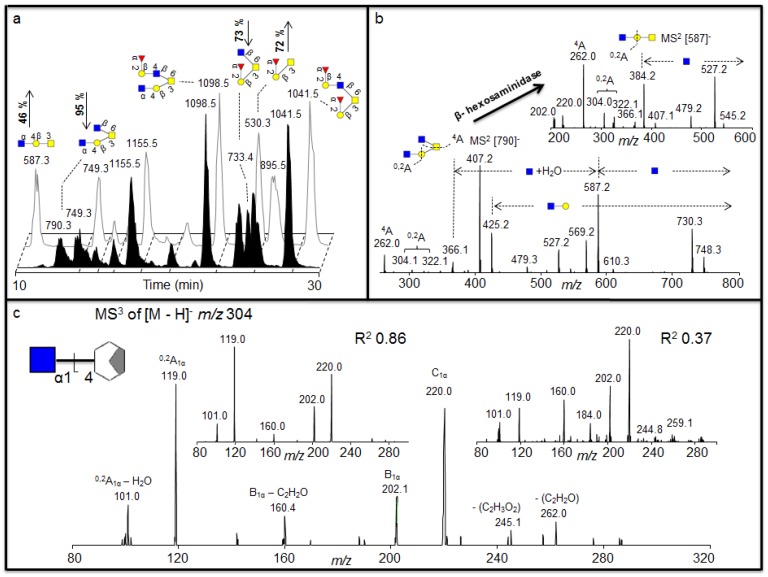
(a) Negative ion baseline chromatograms of β-*N*-acetylhexosaminidase untreated (front) and treated (back) porcine gastric mucin (PGM) oligosaccharides showing the increase of the ions *m/z* 530 and 587 and a decrease of the *m/z* 790 and 733 after treatment. (b) MS^2^ of the [M - H]^-^ ions at *m/z* 790 suggests two terminal HexNAc before treatment and MS^2^ of the [M - H]^-^ ions at *m/z* 587 suggests a core 1 with one terminal HexNAc left after the treatment suggesting the terminal α1-4 linked GlcNAc.(c) The MS^3^ fragmentation of the ^0,2^A_1α_ – H_2_O is shown (bottom) containing the terminal GlcNAc1-4 moiety plus part of the cleaved Gal. Insert shows MS^3^ fragmentation of the ^0,2^A_1α_ – H_2_O fragment ion of *m/z* 304 isolated from GlcNAcβ1-4GlcNAcβ1-4GlcNAc (left) and GalNAcβ1-4Gal (right), showing with the R^2^ values that the linkage of the residue of the sample corresponds to the GlcNAc1-4 linkage of the standard. The GlcNAc was indicated to be α linkage of the structure as discussed in the text. For explanation of symbols, see legend in Table 1.

**Table 1:** The MS^2^ spectral intensity correlation comparison of the sialylated and neutral structures with spectra reported in the MS^2^ database UniCarb-DB. The sialylated structures did not give a good match due to loss of labile sialic acid leaving behind very little information. The R^2^ value is a measure of how well all peaks and their intensities in an MS^2^ spectrum are matching with the database peak list. Symbols used are according to the Consortium for Functional Glycomics guidelines [20] and was generated by Glycoworkbench [21].*indicates structures that are created after de-sialylation.** indicates structures created after loss of terminal GlcNAc.

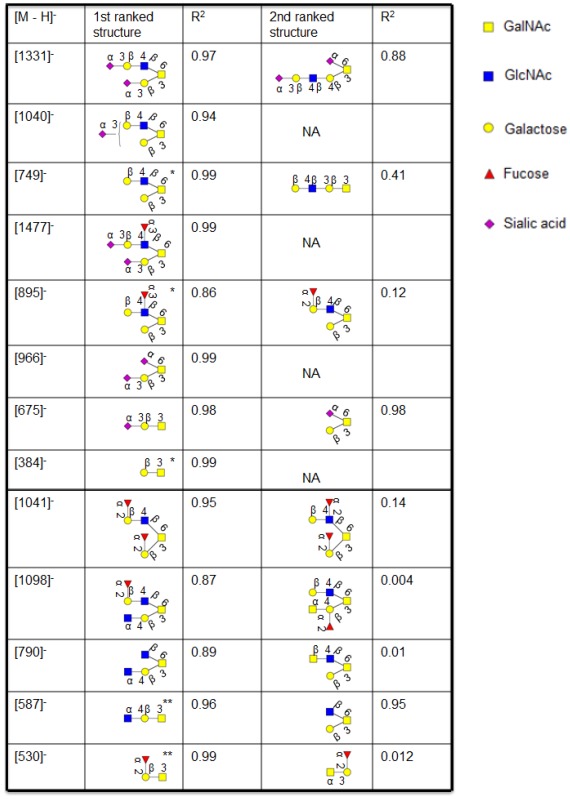

### 2.2. Identification of 4 Linked Terminal GlcNAc Moiety in Porcine Gastric Mucins (PGM)

The exoglycosidase digestion will always be restricted to the availability of specific exoglycosidases. We identified structures in porcine gastric mucin (PGM) oligosaccharides with terminal HexNAc that was not affected by various hexosaminidases [22] (Figure 3), including β- *N*-acetylhexosaminidase from jack bean (JBH, removes both β-linked GlcNAc and GalNAc). We wanted to investigate whether we could further characterize the nature of this terminal HexNAc by LC-MS^2^ and LC-MS^3^. After 16 h incubation with β-*N*-acetylhexosaminidase (specific for β-linked GlcNAc and GalNAc) a substantial drop in intensity of the [M - H]^-^ ions at *m/z* 733 (Fuc_1_Hex_1_HexNAc_1_HexNAcol) and *m/z* 790 (Hex_1_HexNAc_2_HexNAcol) could be identified, accompanied with an increase of the intensity of the [M - H]^-^ ions at *m/z* 530 (Fuc_1_Hex_1_HexNAcol) and *m/z* 587 (Hex_1_HexNAc_1_HexNAcol) (Figure 3a), indicating that these were the exoglycosidase products generated after removal of one HexNAc from each of these substrates, respectively. The MS^2^ spectral intensity correlation analysis of the [M - H]^-^ ions at *m/z* 733 that was degraded suggests a core 2 structure with Fucα1-2Galβ1-3(GlcNAcβ1-6)GalNAc configuration because it gives similar spectra to the spectra reported in the MS^2^ database UniCarb-DB (Table 1). The drop in intensity of the [M - H]^-^ ions at *m/z* 733 after hexosaminidase is due to the degradation of the terminal HexNAc (Figure 3a) generating a core 1 structure terminating in a blood group H epitope (Fucα1-2Galβ1-3GalNAcol) (Figure 3a), which is also supported with spectrum reported in the MS^2^ database UniCarb-DB (Table 1). Hence, this drop in intensity in core 2 sequence Fucα1-2-Galβ1-3(GlcNAcβ1-6)GalNAcα1-Ser/Thr generating core 1 sequence confirmed the terminal HexNAc to be β1-6 linked GlcNAc in the structure. However, the MS^2^ spectral correlation analysis of the [M - H]^-^ ions at *m/z* 790 with spectra reported in the MS^2^ database UniCarb-DB suggests that this was a core 2 structure with HexNAc-Galβ1-3(GlcNAcβ1-6)GalNAc configuration (Table 1) with unknown information about the nature of the HexNAc residue on the C-3 antenna. After hexosaminidase treatment only the C-6 GlcNAc could be removed (Figure 3b). This generated a core 1 structure with one terminal HexNAc still remaining ([M - H]^-^ ions of *m/z* 587) indicating that the second terminal HexNAc was not in a β-configuration (Figure 3b), and treatment with the a-*N*-actetylgalatosaminidase was not successful (data not shown). The MS^2^ spectral correlation analysis of the [M - H]^-^ ions at *m/z* 587 suggests a core 1 structure terminated with HexNAc (Table 1) but did not give conclusive result about the configuration (Table 1) when compared with spectra reported in the MS^2^ database UniCarb-DB. Due to lack of specific enzymes, MS^2^ of the substrate ([M - H]^-^ ions at *m/z* 790) and product ([M - H]^-^ ions at *m/z* 587) were interpreted manually to investigate the configuration of terminal HexNAc (Figure 3b).

The identification of cross ring ^0,2^A fragments of the core 1 GlcNAc residue in the MS^2^ spectra of the substrate at *m/z* 790 and the product at *m/z* 587 (Figure 3b) suggests that this was a terminal HexNAc linked to the 4 position of a Gal because extension to the C-4 provides a diagnostic ion of *m/z* 304 after loss of water, whereas extension of C-3 does not give this fragment [8]. This indicates that the structure of the substrate ([M - H]^-^ ions at *m/z* 790) and product ([M - H]^-^ ions at *m/z* 587) is HexNAc1-4Galβ1-3(GlcNAcβ1-6)GalNAcol and HexNAc1-4Galβ1-3GalNAcol respectively. In addition, the MS^2^ spectral correlation analysis of the [M - H]^-^ ions at *m/z* 1098 that remains undegraded by the treatment with spectra reported in the MS^2^ database UniCarb-DB suggests that this was a core 2 structure (HexNAc-Galβ1-3(Fucα1-2Galβ1-4GlcNAcβ1-6)GalNAcol) terminated with one HexNAc (Table 1). The identification of cross ring ^0,2^A fragments in the MS^2^ spectra of the [M - H]^-^ ions at *m/z* 1098 confirmed that this structure contained 4 linked HexNAc (data not shown). Since this structure was not affected by various β- hexosaminidase digestion suggesting that it contained the α1-4 linked GlcNAc to Gal. 

Having identified that the second terminal HexNAc in the [M - H]^-^ ions at *m/z* 790 is 4 linked, the investigations were extended further to uncover the identity of the 4 linked HexNAc. The literature suggests that some of the terminal 4 linked HexNAc have been identified in the gastric mucin previously. These include the GlcNAcβ1-4GlcNAc chitobiose [23], the antibacterial GlcNAca1-4 motif [24] and the GalNAcβ1-4GlcNAc lacdiNAc motifs [25]. An MS^3^ approach was adopted, whereby the fragmentation pattern of known standards containing GlcNAc1-4 (chitotriose) and GalNAc1-4 (GalNAcβ1-4Gal) were compared to the fragmentation of the 4 linked HexNAc structure identified in the dominating *m/z* 790 isomer in PGM. The cross ring ^0,2^A fragment with an *m/z* 304 characteristic for the C-4 extension of the core 1 HexNAc (Figure 3b) was selected for MS^3^ fragmentation for both the sample and the standards, and the comparison allows assignment of the terminal epitope, since the mechanism for the generation of this fragment [26] removes the anomeric information as well as the stereospecificity of the cross ring fragmentation remnant. Figure 3c shows the spectra for the MS^3^ of PGM with *m/z* 790 parent and subsequent collision of the daughter ion *m/z* 304 and the MS^3^ spectra of the daughter ion *m/z* 304 after collision of the [M - H]^-^ ions for both the standards. 

Correlation of the MS^3^ fragments and their intensities from the PGM sample with the standards showed that standard oligosaccharide with the GalNAc1-4 had an R^2^ value of 0.37; whereas the GlcNAc1-4 had an R^2^ value of 0.86. This data confirms that the second terminal HexNAc in the [M - H]^-^ ions of *m/z* 790 in PGM is 4- linked GlcNAc. However, this data does not suggest the β-configuration due to loss of the anomeric configuration in the ^0,2^A_1α_ - H_2_O fragment ion [26]. The terminal 4 linked GlcNAc was not affected by hexosaminidases digestion, which removes the β 2, 4 and 6 linked GlcNAc and a 3HexNAc, indicating that this terminal GlcNAc is α1-4 linked. Thus, MS^3^ can be used as an alternative, when the lack of exoglycosidases does not allow the assignment of non-reducing monosaccharide moieties. The inability for digestion of this particular terminal HexNAc with currently available *N*-acetylhexosaminidases of known specificity in combination with MS^3^ suggested that the [M - H]^-^ ions at *m/z* 790 in PGM contains the antibacterial terminal α1,4 linked GlcNAc epitope [24]. 

### 2.3. Investigating the Nature of Endogenous Salivary Exoglycosidase Digestion Using UniCarb-DB

**Figure 4:**
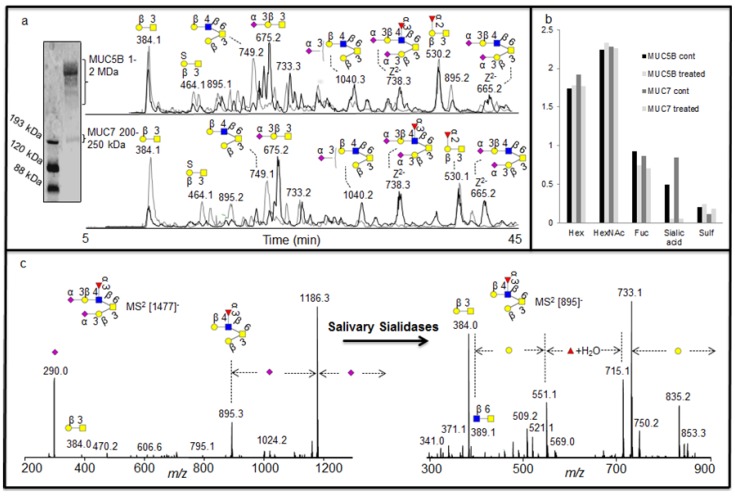
(a) Enrichment of salivary glycoproteins MUC5B and MUC7 by SDS-AgPAGE with their negative ion baseline chromatograms of MUC5B and MUC7 oligosaccharides before (front black) and after (back grey) the treatment with saliva. (b) The average composition of monosaccharide in the untreated and treated sample based on MSAC. (c) MS^2^ of the [M - H]^- ^ions at *m/z* 1477 before treatment and [M - H]^-^ ions at *m/z* 895 created after the treatment for sequence elucidation.

Saliva is known to contain endogenous exoglycosidase activity, mainly due to the presence of salivary bacterial secretion of exoglycosidases, which digest complex oligosaccharides into monosaccharide units as a source of nutrients. Since our approach using MS^2^ database fragment matching alongside exoglycosidase as illustrated above, generated very detailed information about oligosaccharide sequences, it was also investigated whether this approach could identify the specificity of exoglycosidases present in saliva. Investigation of complex degradation patterns of mucin oligosaccharides in biological fluids involves a mixture of glycosidases and their effect on a spectrum of oligosaccharides. The salivary mucins MUC5B and MUC7 were isolated by SDS-AgPAGE (Figure 4a) and blotted onto PVDF membranes. The blots were treated with saliva and control saliva (saliva boiled for 15 minutes) isolated from a healthy individual. The blots were washed and oligosaccharides were released by reductive β-elimination and analyzed by LC-MS [18]. The structures identified with and without the salivary treatments were assigned by comparison of MS^2^ spectral intensity correlation with spectra reported in the MS^2^ database UniCarb-DB [16]. The assignment of the structures showed that the untreated samples were highly sialylated, while the increase in the intensity of neutral structures after treatment suggested that these were the exoglycosidase products generated after removal of sialic acid (Figure 4a). This indicated that either there is an endogenous sialidase or a battery of sialidases, with similar specificities from different micoorganisms, is responsible for most of the exoglycosidase activity in saliva. This was also suggested by the average composition (MSAC= mass spectrometric average composition, [27]) of the oligosaccharide based on the mass spectrometric intensities (Figure 4b).

As was shown with the synovial lubricin sialylation, there were few linkage-specific fragments available in the MS^2^ fragments of sialylated structures. The spectra were also dominated by the loss of sialic acid from the parent ion (Figure 4c). The nature of the fragmentation of sialylated structures made some of the MS^2^ spectral intensities not decisive when compared with spectra reported in the MS^2^ database UniCarb-DB (Table 1), while after salivary sialidase, the spectra of neutral oligosaccharides include information about core and linkage type as well as the nature of fucose substitution [8] and better scoring with spectral matching. This fact is illustrated in figure 4c and Table 1, where the MS^2^ spectral intensity correlation comparison of the [M - H]^-^ ions at *m/z* 895 (Fuc_1_Hex_2_HexNAc_1_HexNAcol), which is the desialylated product of *m/z* 1477 (NeuAc_2_Fuc_1_Hex_2_HexNAc_1_HexNAcol) with spectra reported in the MS^2^ database UniCarb-DB suggests that this was a core 2 structure with Galβ1-3(Galβ1-4(Fucα1-3)GlcNAcβ1-6)GalNAcol configuration (Table 1) which can be terminated with one sialic acid (on either of the branches) and with two sialic acid (on both branches). The branching as a Lewis x type structure (Galβ1-4(Fucα1-3)GlcNAcβ1-) is indicated by the Z/Z and Z/Z - MeOH fragment pair of *m/z* 551 and 521 [5].

**Figure 5:**
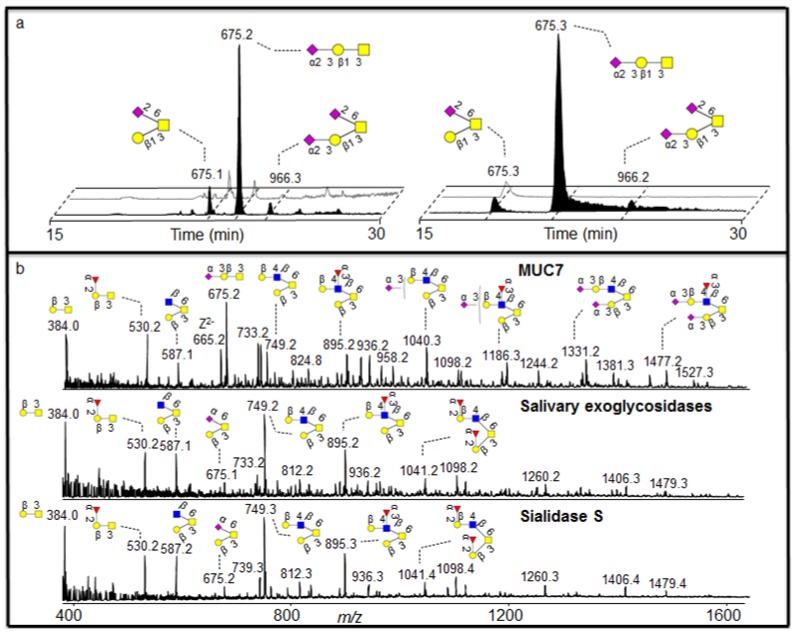
Linkage specific sialidase activity of saliva. (a) SIC of *m/z* 675 and 966 before (front) and after (back) incubation with saliva (left) and sialidase S (right) showing linkage specific sialidase activity of saliva. (b) Negative ion MS profile of MUC7 oligosaccharides before and after treatment with saliva and sialidase S. For explanation of symbols, see legend in Table 1.

In order to further characterise the nature of the salivary sialidase, we were guided by the fact that salivary MUC7 has been shown to be dominated by 3 linked sialic acid [18]. Indeed, treatment of MUC7 oligosaccharides with sialidase S (specific for α2-3 sialic acid) generated an oligosaccharide profile similar to the saliva treatment (Figure 5b). 

In order to identify if the salivary sialidase were specifically included in 3 linked sialic acid, we were able to identify two components in the MUC7 sample, where 6 linked sialic acid was also present. Interpretation of low abundant fragment ions of the earlier eluting isomer with the MS^2^ of the [M - H]^-^ ions at *m/z* 675 showed that it was core 1 with sialic acid linked to HexNAcol because it generated a glycosidic Y fragment ion at *m/z* 513 losing a terminal Hex. This makes a sequence identical to a galactosylated sialyl-Tn structure (Galβ1-3(NeuAcα2-6)GalNAcol. The low abundant [M - H]^-^ ions of *m/z* 966 is the extension of this structure and one additional 3 linked sialic acid attached to the C-3 linked galactose (Figure 5a, left). In Figure 5a (left), the late eluting singly sialylated core 1 isomer with [M - H]^-^ ions of *m/z* 675 with 2-3 linked sialic acid was completely degraded while the early 2-6 linked isomer remained virtually undegraded. The intensity of the low abundant [M - H]^-^ ions of *m/z* 966 was also lowered, possibly degraded and detected as the small increase of the early eluting *m/z* 675 isomer. The degradation of the 2-3- linked sialic acid is accompanied by an increase in the intensity of core 1 (data not shown), which is created by the removal of sialic acid. This linkage specific desialylation of saliva is supported by sialidase S treatment of MUC5B and MUC7 (Figure 5a right). As discussed earlier, the MS^2^ spectral intensity correlation comparison of the sialylated structures did not give decisive results. Hence, manual interpretation of the MS^2^ fragmentation was necessary for assigning sialic acid linkage. 

### 2.4. Discussion

The availability of specific exoglycosidases, alongside the spectral library of *O*-linked oligosaccharide collision induced dissociation (CID) MS fragmentation, as a method for structural assignment of oligosaccharide structures was determined by incubating human synovial lubricin with sialidase S. Lubricin is a mucin like glycoprotein with extensive *O*-linked glycosylation. The abundance of negatively charged glycans of lubricin contributes to the proteins boundary lubrication of the cartilage surface due to strong repulsive hydration forces [28,29,30,31]. During inflammation, the glycosylation properties such as sialylation, fucosylation and sulfation are regulated to manipulate cell adhesion, differentiation, maturation and activation in the case of immune cells. The literature [28,32] suggests that glycosidases such as galactosidases and neuraminidases significantly reduce the lubricating property of lubricin. Before incubation with sialidase S, the MS^2^ spectral intensity of the sialylated structure gave an indecisive result when compared with spectra reported in the MS^2^ database UniCarb-DB. The incubation of human synovial lubricin with sialidase S indicates the degradation of mono-sialylated core 1 and mono- and di-sialylated core 2 structures (Figure 2b), which is accompanied by an increase in the intensity of the neutral structures generated by the removal of sialic acid (Figure 2b). The MS^2^ spectral intensity correlation with spectra reported in the MS^2^ database UniCarb-DB helped in assigning the structure created by the removal of sialic acid, while the degradation of these mono-sialylated core 1 and mono- and di-sialylated core 2 structures are terminated by α2-3 –linked sialic acid. The exoglycosidase digestion specific to sialic acid and a MS^2^ spectral library comparison minimized the use of time-consuming exoglycosidase digestion to monosaccharide unit for structural assignment. This degradation suggested that these mono-sialylated core 1 and mono- and di-sialylated core 2 structures are terminated by α2-3 –linked sialic acid. 

Having shown that exoglycosidase digestion of human synovial lubricin oligosaccharides and a MS^2^ spectral library comparison can provide information about assignment of individual structures present in the sample, we extended our analysis into addressing the assignment of the non-digestible terminal HexNAc configuration present in PGM oligosaccharides using MS^3^. This suggested that the non-digestable terminal HexNAc in PGM oligosaccharides may be the antibacterial terminal α1,4 linked GlcNAc epitope.

In order to address the exoglycosidase activity of saliva we proposed that saliva is capable of digesting mucin oligosaccharides still attached to mucins blotted onto pvdf membranes. The human oral cavity sustains the growth of more than 500 different strains of bacteria [33] of which both harmful and beneficial bacteria use the oligosaccharide chains of mucins as a nutrient source [34]. Oral bacteria such as *Bacteroides forsythus*, *Actinobacillus*, *Actinomycetemcomitans* and *Porphyromonas gingivalis* are shown to be associated with peridontitis [10,35] while other bacteria cause root canal infections [36]. The high molecular weight mucins with their high degree of *O*-linked glycosylation (50–80% of total weight) in their Ser/Thr/Pro rich domains [37] is involved in protection against oral bacteria. There is growing evidence that shows that mucin glycosylation can change in response to mucosal infection and inflammation [2]. This will alter the oral milieu for the bacteria and how they interact with oral surfaces. Bacteria will degrade oligosaccharides from mucins in order to make them available as a nutrient source [38,39,40]. This degradation is achieved by the production of glycosidases such as; α-*N*-acetyl-D-galactosamindase, sialidase, β-galactosiminidase, β-*N*-acetlyglucosaminidase, α-and β-mannosidase, and α-fucosidas [41,42]. The results from salivary MUC5B and MUC7 after incubation with saliva indicate high level of sialidase activity under the conditions applied. The removal of sialic acid makes new monosaccharide units accessible for salivary exoglycosidases. Hence, this step is important to enable the degradation of salivary mucins. Preliminary data showed that sialidases and proteases work in parallel to degrade the mucins (data not shown), indicating that sialidase not only exposes new oligosaccharide epitopes for further exoglycosidase digestion, but also makes the protein backbone more accessible for proteolytic degradation. The literature suggests that the exposure of the mucin protein backbone (mucins expressed in the intestine) to proteolytic enzymes produced by various bacteria [43] may result in the host becoming more prone to infections, as shown in the cases of ulcerative colitis and Crohn’s disease [44]. However, the degradation of oral mucins is complex, requiring multiple strains of bacteria to co-exist in a symbiotic relationship [45]. Some bacteria produce enzymes that degrade the oligosaccharide side chains of mucins, while others produce proteolytic enzymes [45]. To understand this relationship, measuring the combined effect of multiple exoglycosidases on multiple oligosaccharide epitopes will provide clues into distinguishing the conditions provided by commensal bacteria from pathological conditions.

## 3. Experimental Section

### 3.1. Materials and Methods

The sialidase S/NANase I (recombinant from *Streptococcus pneumonia*, expressed in *E. coli*), glyko β-*N*-acetylhexosaminidase (jack bean)/HEXase III, β-*N*-acetylglucosaminidase (GUH) were obtained from Prozyme Co. (Hayward, CA, USA) and α-*N*-acetylgalactosaminidase from *C. perfringens* was obtained from R&D systems (Minneapolis, MN, USA). PGM, dithiothreitol (DTT) and iodoacetamide (IAA) were obtained from Sigma Aldrich Co. (St Louis, MO, USA). Hypersep hypercarb SPE columns (60106-301) were obtained from Thermo Scientific Co. (Sanford, FL, USA). The NuPAGE gels were obtained from Invitrogen Co. (Grand Island, NY, USA).

### 3.2. Enrichment of Salivary Mucins (MUC5B and MUC7) and Synovial Lubricin

Saliva (5 ml) from a donor and synovial lubricin purified from human synovial fluid as described previously [8] were collected, reduced in NuPAGE sample buffer containing dithiothreitol (10 mM) for 20 minutes at 80 °C and alkylated with 25 mM of iodoacetamide for 1h in the dark [6]. The saliva (25 µL) and synovial lubricin (10 µg) sample were then loaded onto a SDS-polyacrylamide/agarose composite gel (0-7%) [6] and SDS-PAGE gel (3-8%) respectively. The SDS-AgPAGE were run in boronate/Tris buffer (192 mM boric acid, 1 mM EDTA, pH adjusted to 7.6 with Tris and 0.1% SDS) and SDS-PAGE were run in Tris acetate buffer (0.1 M Tricine, 0.1 M Tris pH adjusted to 8.4 and 0.1% SDS) respectively until the dye front ran out of the gel. The gels were blotted to PVDF membranes, stained with alcian blue, and oligosaccharides were released by reductive β-elimination as described previously [6,10]. 

### 3.3. Exoglycosidase Treatment and Release of O-Linked Oligosaccharides

Oligosaccharide mixture from PGM (10 µg) (Sigma-Aldrich, St Louis, MO, USA) were digested in 0.5 mU jack bean/HEXase III or GUH hexosaminidase or α- *N*-acetylgalactosaminidase in 10 µL of reaction buffer for 1h at 37 °C. Purified human synovial lubricin oligosaccharides from synovial fluid were digested in 0.5 mU sialidase S/NANase I in 10 µL of reaction buffer for 16 h at 37 °C. Salivary mucins (MUC5B and MUC7) blotted onto PVDF membranes after SDS-AgPAGE (Figure 4a) were incubated with fresh saliva and control saliva (saliva boiled for 15 min) for 6 h at 37 °C. For confirmation of linkage specific sialidases, released MUC5B and MUC7 oligosaccharides were digested in 0.5 mU sialidase S/NANase I in 10 µL of reaction buffer for 16h at 37 °C

Salivary mucin oligosaccharides were released for exoglycosidase activity LC-MS monitoring by reductive β-elimination as described previously [6,10]. Monitoring of exoglycosidase using already released oligosaccharides was performed after desalting using graphitized carbon packed in micro zip-tips as described [10].

### 3.4. LC-MS^2^ and LC-MS^n^ Analysis of Oligosaccharides and Interpretation of Data

Sample injection and LC was performed by using a CTC PAL autosampler and LC pump (Agilent, Santa Clara, CA, USA). Oligosaccharides were analyzed by capillary graphitized carbon(10 × 0.25 mm id, 5 µm Hypercarb particles, Thermo-Hypersil, Runcorn, UK) LC-MS and LC-MS^2^ in negative ion mode using an LTQ mass spectrometer (Thermo-Fisher, San Jose, CA, USA). Oligosaccharides were eluted with an H_2_0/acetonitrile gradient containing 10 mM NH_4_HCO_3_ (0–35% acetonitrile in 45 min, 10 min wash with 100% acetonitrile and 15 min equilibration with 0% acetonitrile). The capillary voltage and the spray voltage for the mass spectrometer were set to 3 V and 2.6 kV respectively and the capillary temperature was set to 300 °C. Air was used as a sheath gas and a full scan ranges from *m/z* 380 to *m/z* 2000 were defined for the structures to be analyzed. Specified ions were isolated for MS^2^ and MS^n^ fragmentation (mass window of 2 Da) by collision induced dissociation (CID) with the collision energy set to 35% and activation time to 30 mseconds. The degradation resistant structure Fucα1-2Galβ1-3(Fucα1-2Galβ1-GlcNAcβ1-6)GalNAcol with an [M - H]^-^ ions of *m/z* 1041, Fucα1-2Galβ1-3GalNAcol with an [M - H]^-^ ions of *m/z* 530 and the sialidase resistant lactone of sialylated core 1 (NeuAcα2-3Galβ1-3GalNAcol) with an [M - H]^-^ ions of *m/z* 657 were used as an internal standard for porcine gastric mucin, salivary mucin and synovial lubricin oligosaccharide, respectively. For structural assignment using MS^2 ^spectral matching, the relative intensity from each *m/z* value from the UniCarb-DB database peak list (www.unicarb-db.com) was downloaded for each structure with the same composition as the unknown. This intensity was matched with the corresponding relative intensity in the MS^2^ spectra of the unknown within 0.5 Da. In order to perform the comparison the sample peak lists were centroided using the Qual Browser 2.07 (Thermo-Fisher) module. The matching exercise was performed manually using an excel spread sheet containing MS^2^ peak lists from unknowns and from the database. The R^2^ value (coefficient of determination) based on linear regression between matched intensity levels of MS^2^ spectra of unknown and from database was used to score each match.

In order to evaluate the amount of degradation of the oligosaccharides during the release (also known as peeling), major degradation products arising from the labile C-3 branch of GalNAc were monitored. The expected peeling products NeuAcα2-3Gal at *m/z* 470 (unreduced) and *m/z* 472 in negative ion mode were found to be close to the baseline, which indicates negligible amount of glycan degradation during release.

A GlcNAcβ1- 4GlcNAc β1- 4GlcNAc standard (Sigma Aldrich, St Louis, MO) and GalNAcβ1- 4Gal standard (DextraUK, Reading, UK) were used to obtain the fragmentation spectra of a terminal 1- 4 linked GlcNAc and a 1- 4 linked GalNAc. 

## 4. Conclusions

Combining LC-MS^2^ spectral matching of oligosaccharide fragment databases with exoglycosidase treatment and salivary exoglycosidase digestion provide an excellent approach for the structural characterization of *O*-linked oligosaccharides. This approach also allows the determination of the nature of exoglycosidases from biological fluids and may help in understanding effective protection against pathological and commensal bacteria. 
